# Supplementary figures and images for: Diet modulates strongyle infection and microbiota in the large intestine of horses
Source: PLoS One. 2024 Apr 9;19(4):e0301920. doi: 10.1371/journal.pone.0301920 (PMC11003623; doi:10.1371/journal.pone.0301920)

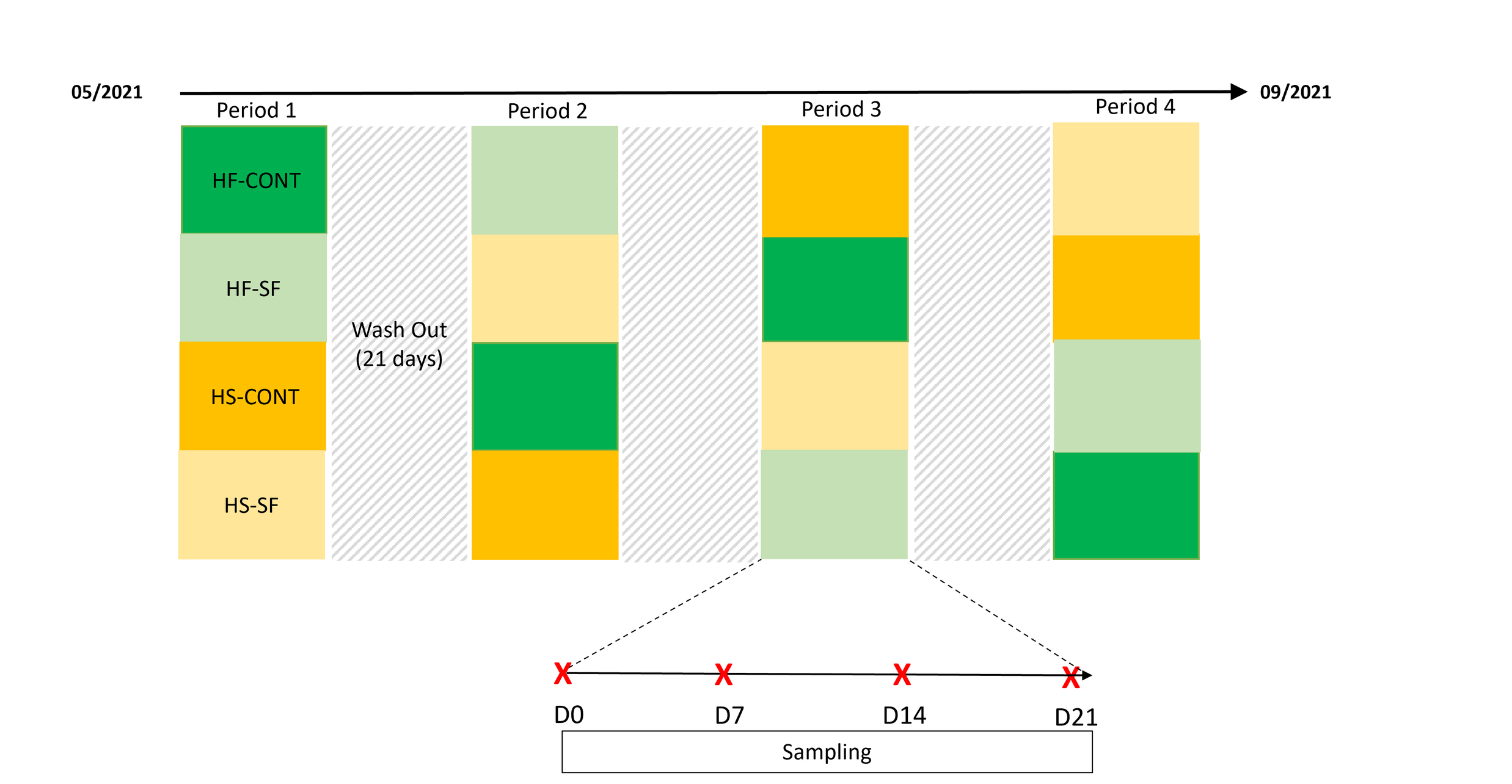

Supplement: S1 Fig — Latin square design with four experimental periods of 21 days and 21 days of wash out between periods. (HF: high-fiber diet; HS: high-starch diet; SF: sainfoin pellets supplementation; CONT: control pellets supplementation). (TIF) [file pone.0301920.s001.tif]

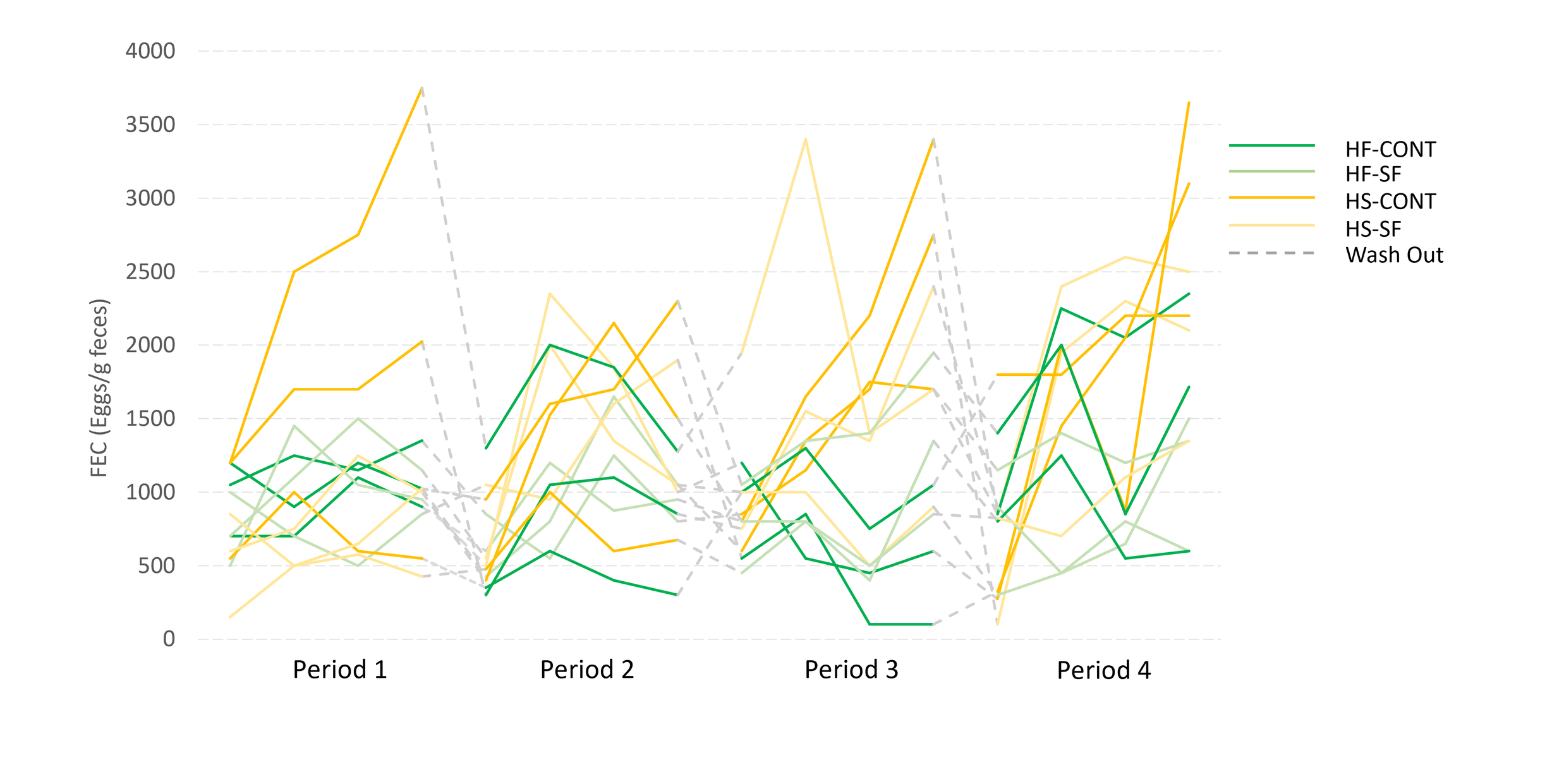

Supplement: S2 Fig — The different colors represent the diet allocated to each horse at a specific period. (HF: high-fiber diet; HS: high-starch diet; SF: sainfoin pellets supplementation; CONT: control pellets supplementation). (TIF) [file pone.0301920.s002.tif]

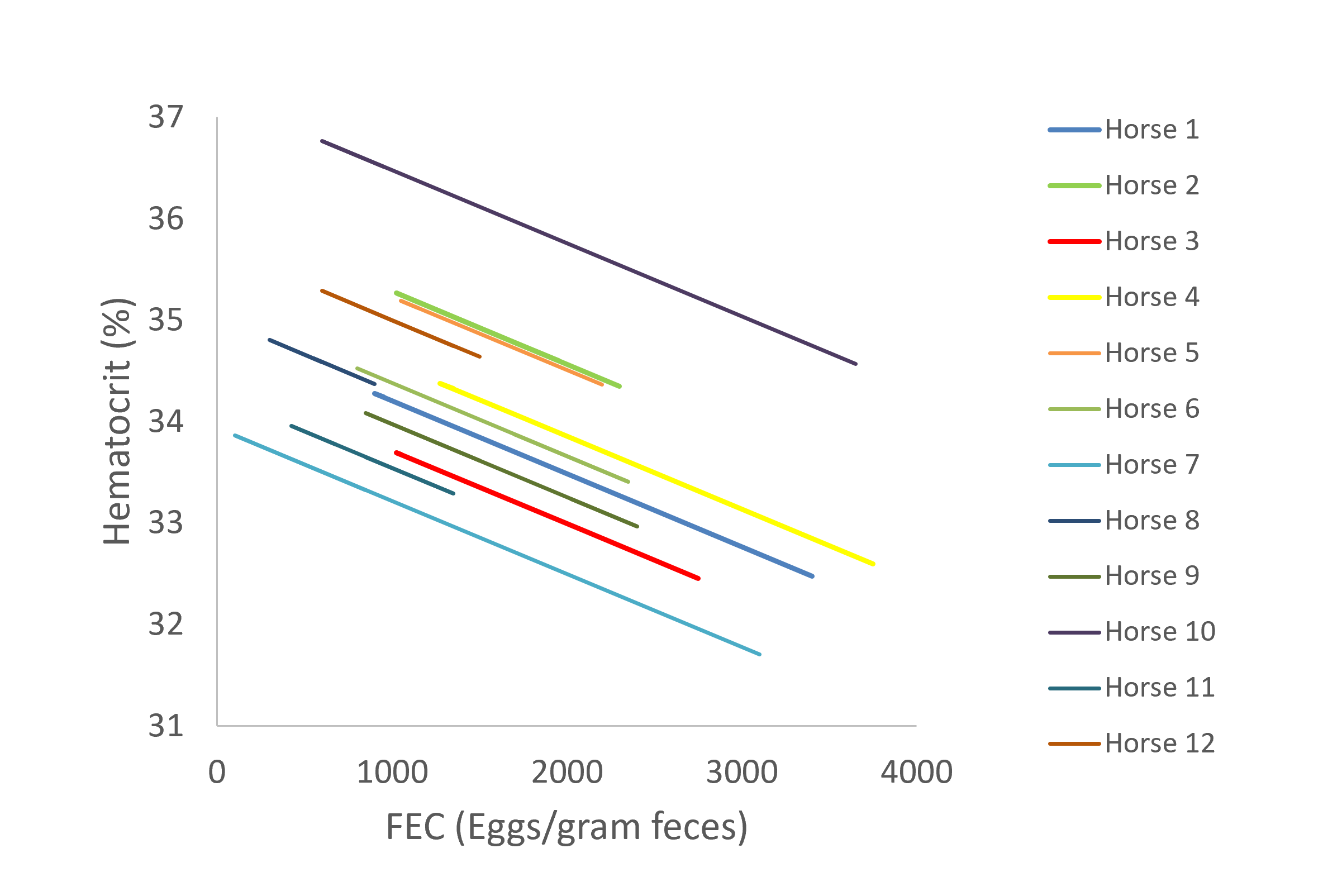

Supplement: S3 Fig — Predicted values from a GLMM that that included the effect of the diet, the supplementation and the FEC as fixed effects and horse ID and horse ID * experimental period as random effects. (TIF) [file pone.0301920.s003.tif]

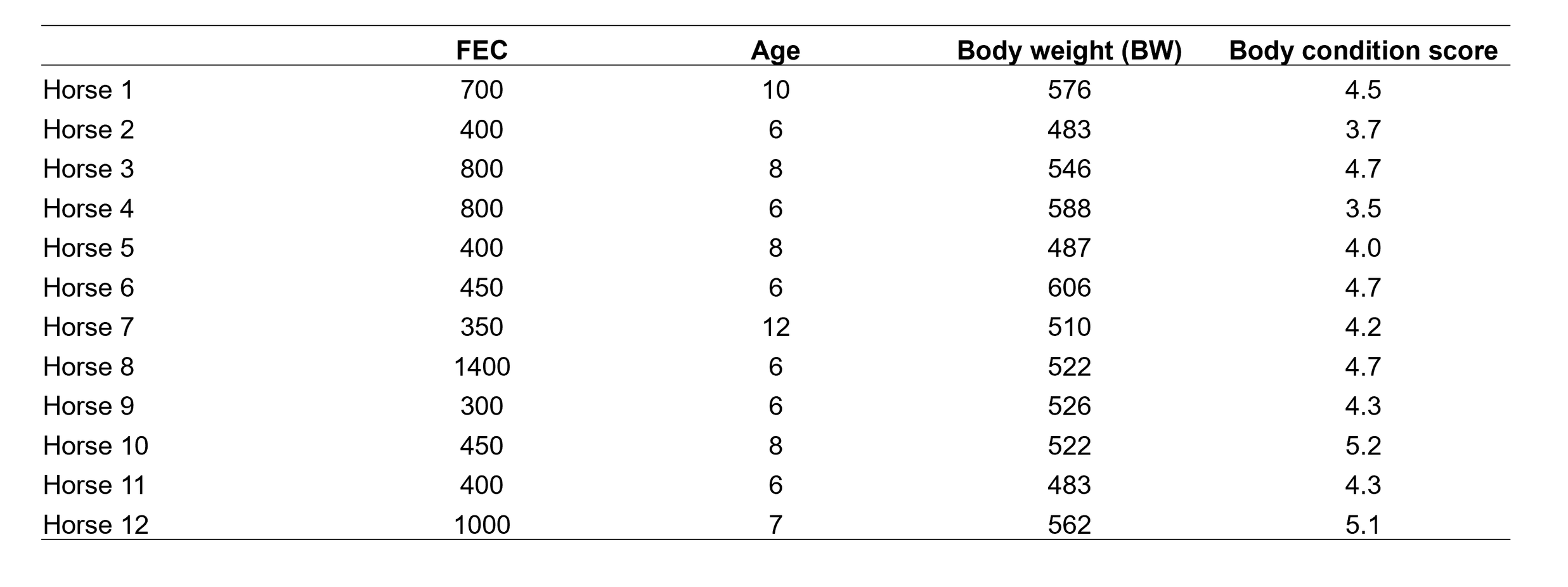

Supplement: S1 Table — (TIF) [file pone.0301920.s004.tif]

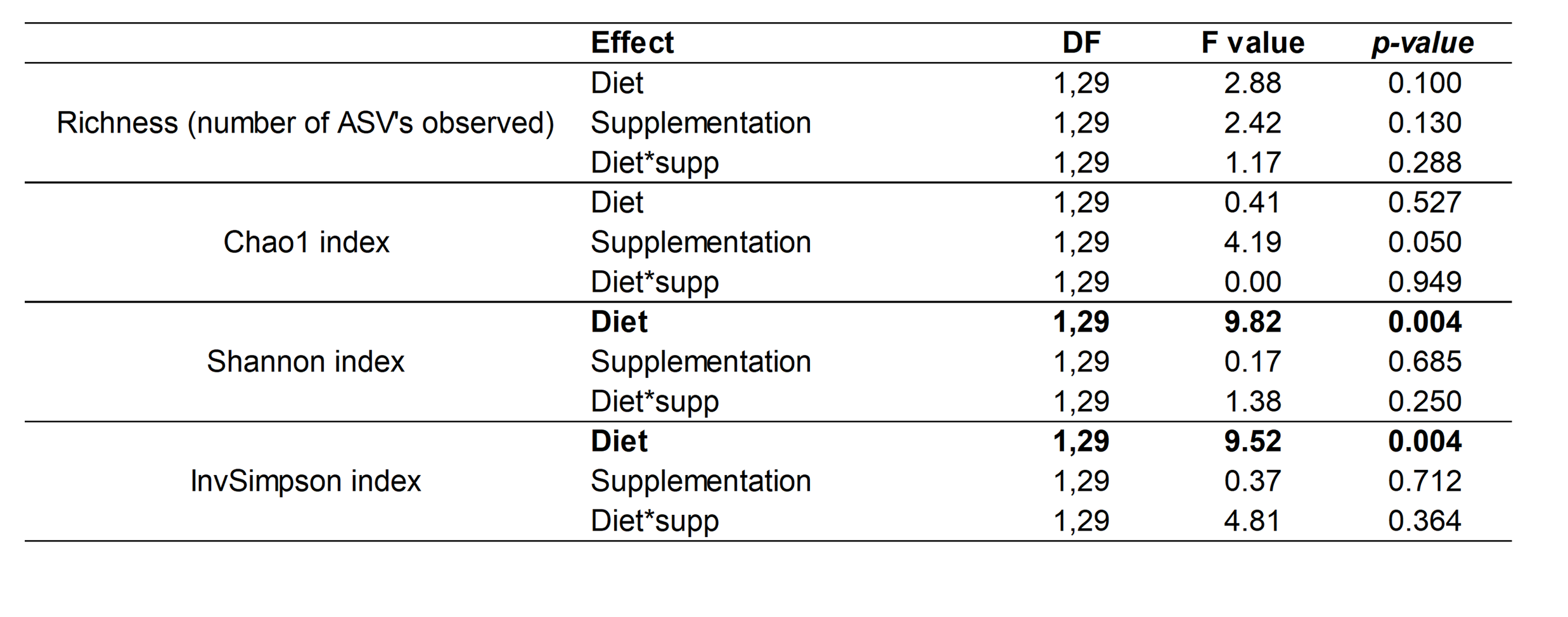

Supplement: S2 Table — We report the fixed effects, but the model also included the horse ID and the horse ID * experimental as random effects. We report the degrees of freedom (df), F and p values. (TIF) [file pone.0301920.s005.tif]

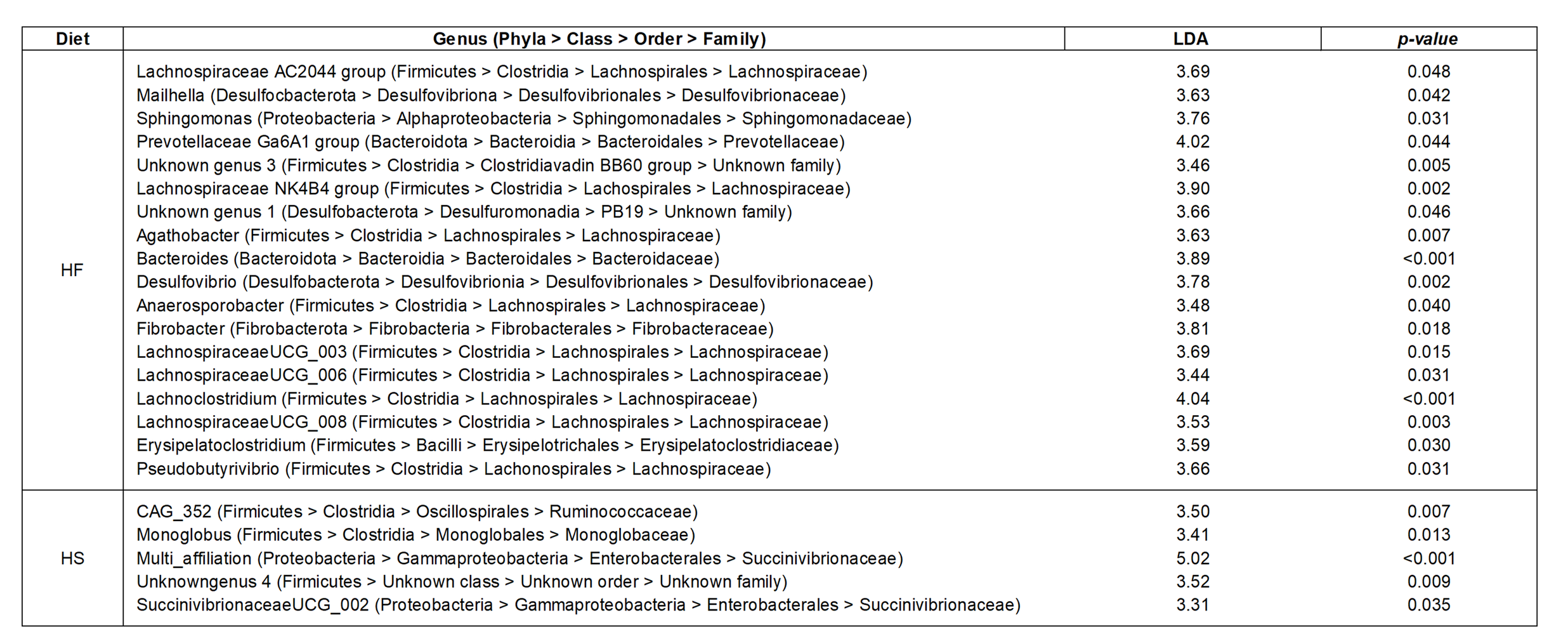

Supplement: S3 Table — (TIF) [file pone.0301920.s006.tif]

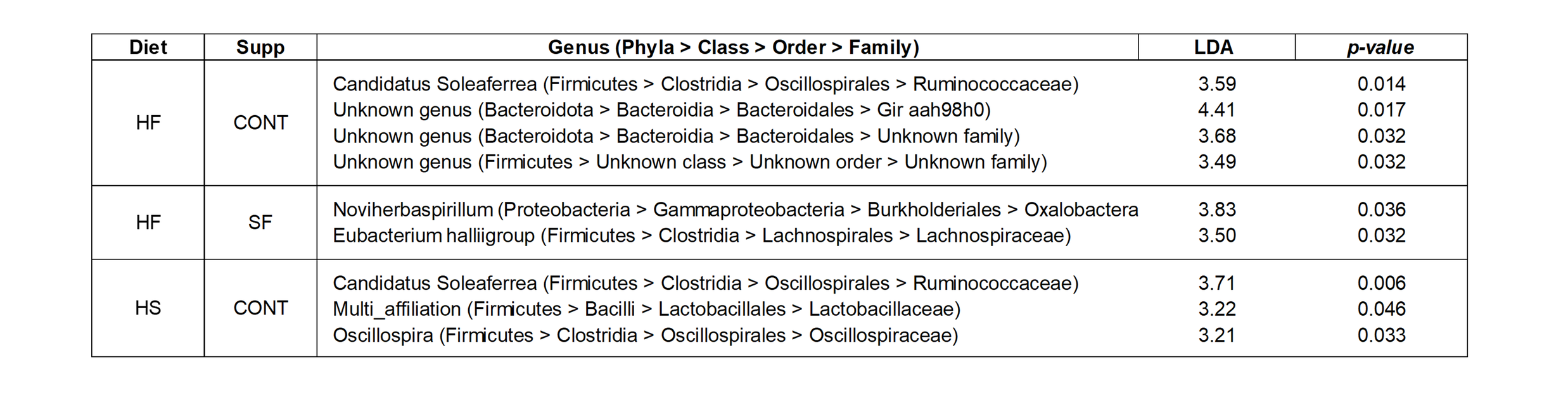

Supplement: S4 Table — (TIF) [file pone.0301920.s007.tif]

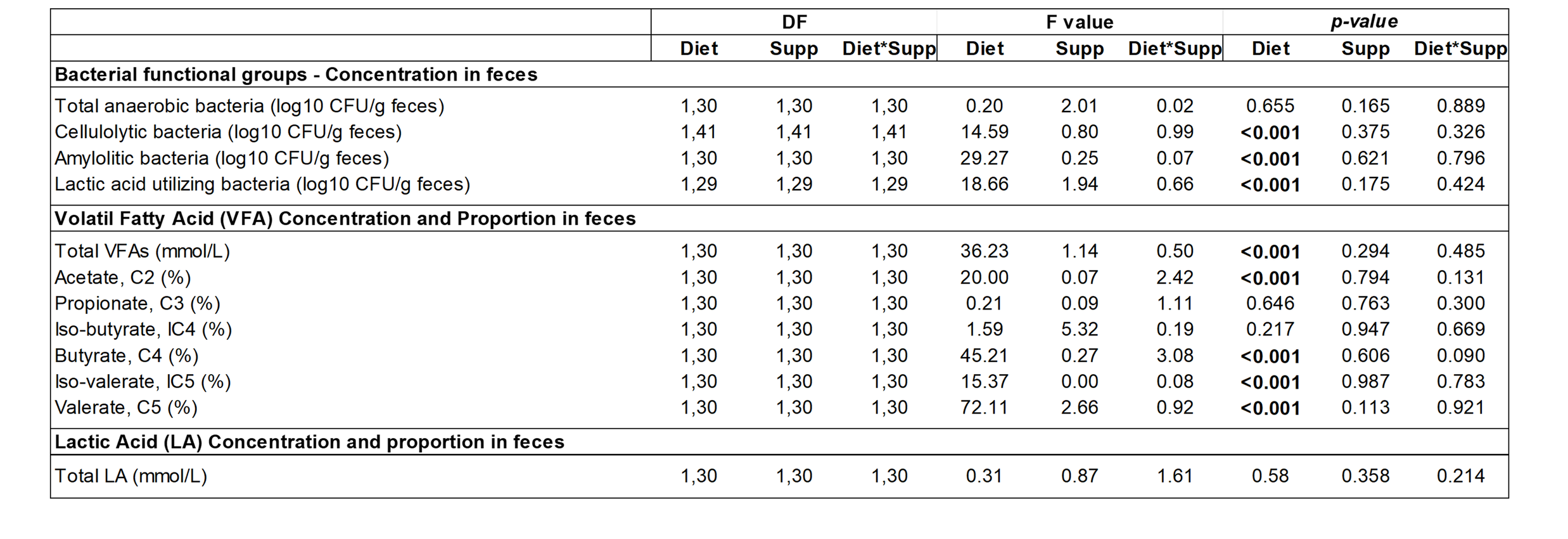

Supplement: S5 Table — We report the fixed effects but the model also included the horse ID and the horse ID * experimental. We report the degrees of freedom, F and p values. (TIF) [file pone.0301920.s008.tif]

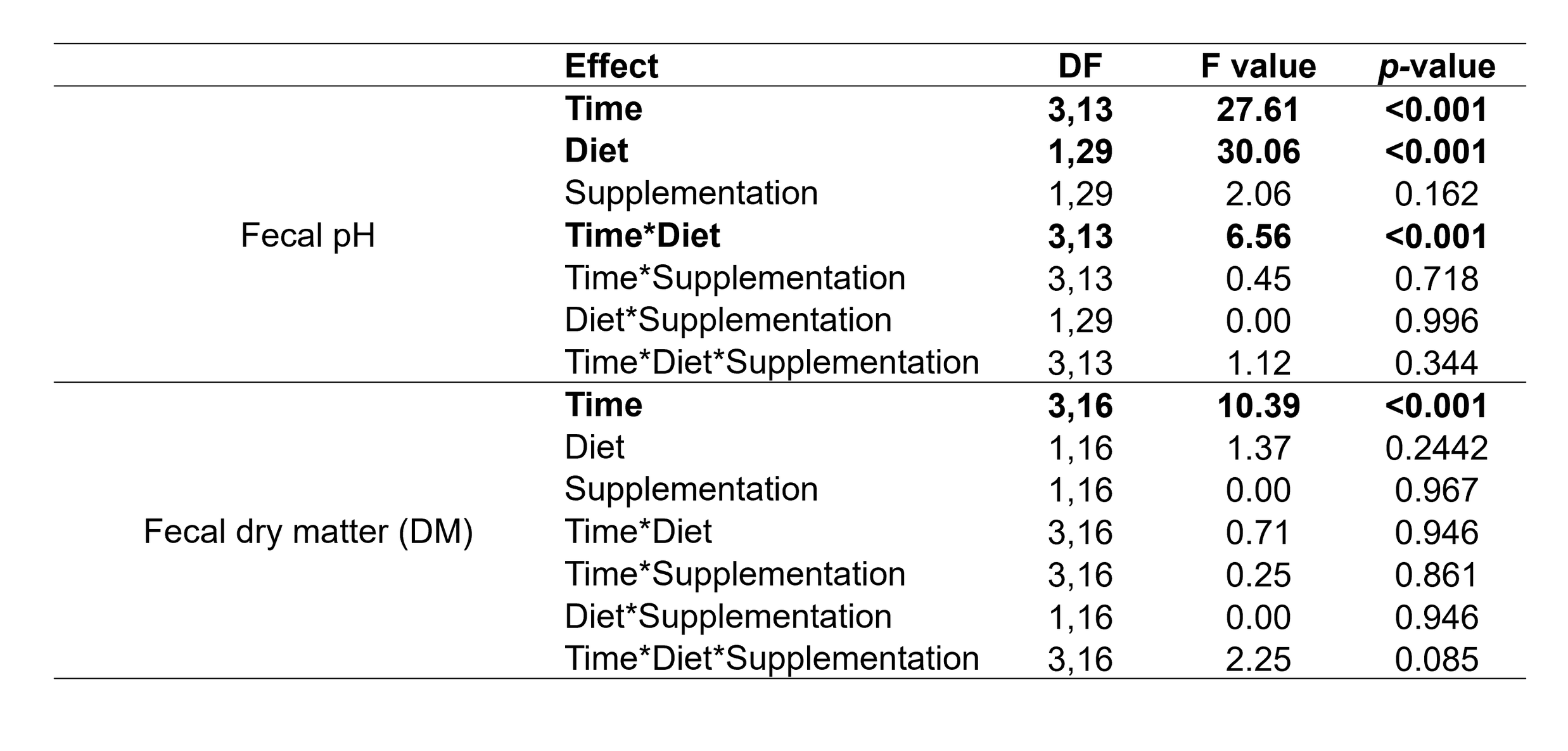

Supplement: S6 Table — We report the fixed effects, but the model also included the horse ID and the horse ID * experimental period as random effects. We report the degrees of freedom (df), F and p values. (TIF) [file pone.0301920.s009.tif]

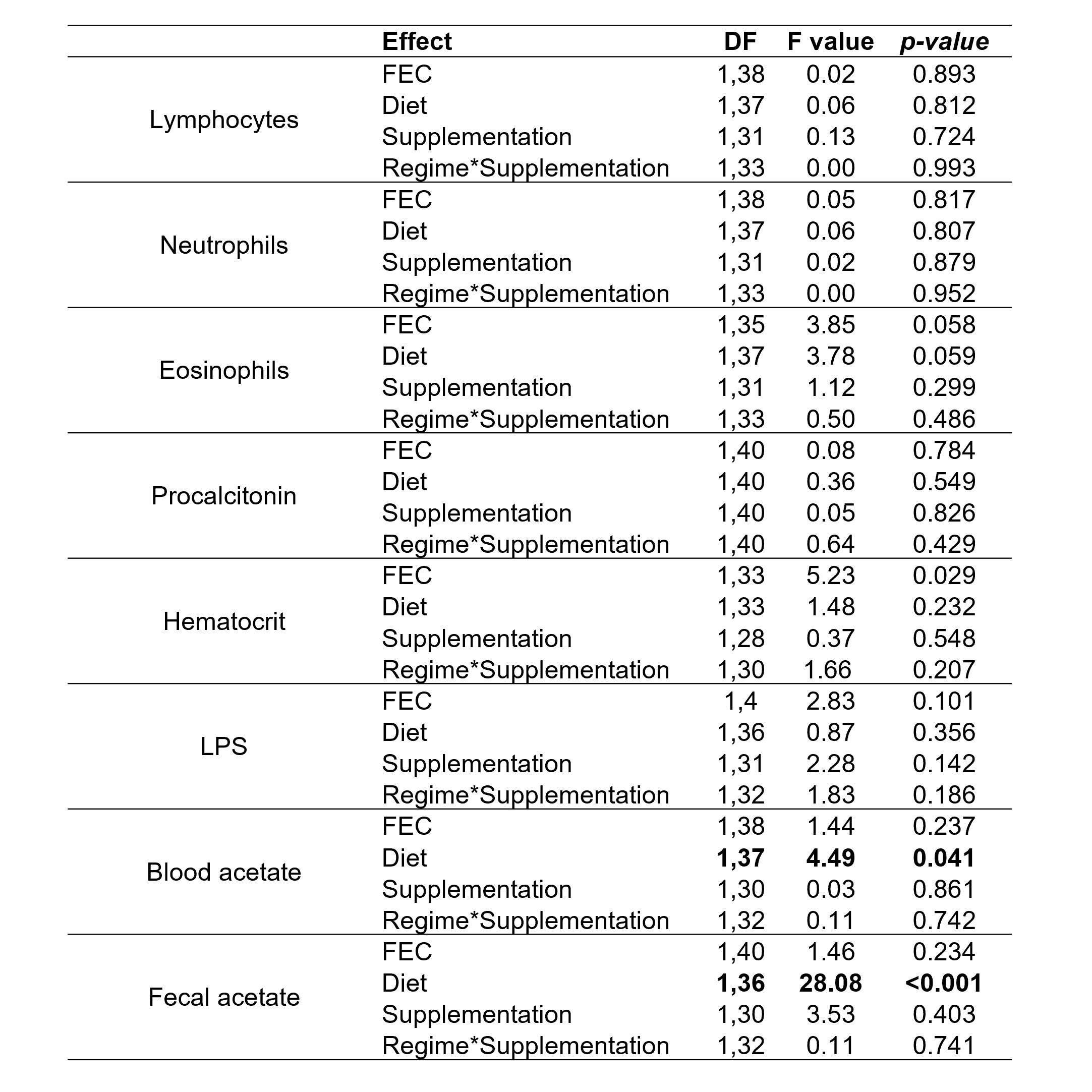

Supplement: S7 Table — We report the fixed effects, but the model also included the horse ID and the horse ID * experimental period as random effects. We report the degrees of freedom (df), F and p values. (TIF) [file pone.0301920.s010.tif]
