# Supplementary material for: Diet modulates strongyle infection and microbiota in the large intestine of horses
Source: PLoS One. 2024 Apr 9;19(4):e0301920. doi: 10.1371/journal.pone.0301920 (PMC11003623; doi:10.1371/journal.pone.0301920)
Supplement: Attachment — Submitted filename: Response to Reviewers.pdf [file pone.0301920.s011.pdf]

Rebuttal letter

Dear editor and reviewers,

We would like to thank you for reviewing our manuscript and giving us relevant and valuable comments to improve it. We address a point-by-point response in table below.

1. Please ensure that your manuscript meets PLOS ONE's style requirements, including those for file naming. The PLOS ONE style templates can be found at

https://journals.plos.org/plosone/s/file?id=wjVg/PLOSOne_formatting_sample_main_body.pdf

and

https://journals.plos.org/plosone/s/file?id=ba62/PLOSOne_formatting_sample_title_authors_affiliations.pdf

Authors' answers: Done.

2. In your Methods section, please provide additional information regarding the permits you obtained for the work. Please ensure you have included the full name of the authority that approved the field site access and, if no permits were required, a brief statement explaining why.

Authors' answers: We conducted the study in the experimental stables of our company, Lab To Field. We added this information in the manuscript at line 131.

3. Thank you for stating the following in your Competing Interests section:

"The authors declare no conflict of interest. The funders had no role in the design of the study, the collection, analysis or interpretation of the data; in the writing of the manuscript, or in the decision to publish the results."

Please complete your Competing Interests on the online submission form to state any Competing Interests. If you have no competing interests, please state "The authors have declared that no competing interests exist.", as detailed online in our guide for authors at http://journals.plos.org/plosone/s/submit-now

This information should be included in your cover letter; we will change the online submission form on your behalf.

Authors' answers: Done.

4. PLOS requires an ORCID iD for the corresponding author in Editorial Manager on papers submitted after December 6th, 2016. Please ensure that you have an ORCID iD and that it is validated in Editorial Manager. To do this, go to 'Update my Information' (in the upper left-hand corner of the main menu), and click on the Fetch/Validate link next to the ORCID field. This will take you to the ORCID site and allow you to create a new iD or authenticate a pre-existing iD in Editorial Manager. Please see the following video for instructions on linking an ORCID iD to your Editorial Manager account: https://www.youtube.com/watch?v=_xcclfuvtxQ

Authors' answers: Done.

5. Please review your reference list to ensure that it is complete and correct. If you have cited papers that have been retracted, please include the rationale for doing so in the manuscript text, or remove these references and replace them with relevant current references. Any changes to the reference list should be mentioned in the rebuttal letter that accompanies your revised manuscript. If you need to cite a retracted article, indicate the article's retracted status in the References list and also include a citation and full reference for the retraction notice.

Authors' answers: Done.

Reviewer #1

Concerning your manuscript PONE-D-23-42676 Article Type: Research Article "Diet modulates strongyle infection and microbiota in the large intestine of horses", I believe it is a very interesting field of applied parasitology and microbiology of domestic and wild equids, namely because horse's feed/food may promote and/or interfere with both. Besides, since the research on this sense is in need, this paper comes in a good timing, so that countries more prone to horse production can have access to this type of data, since its main conclusions can drive veterinarians, horse producers and owners for a more comprehensive work together, towards a more assertive way of managing horse feeding and its consequences on both gastrointestinal parasitic disease and gut microbiology, which can impact seriously the digestion, gut health and performance. Besides what will be pointed out, namely that your manuscript has potential to be published, the final decision on the publication of your manuscript at PLOS ONE, depends on the Editor final statement.

**Referees' comments**	**Authors' answers**
Introduction<br>Page 3 – You focused too much your attention on the impacts of parasites and their consequences in ruminants. Taken that your manuscript deals with horse parasites, you should write something on this sense, regarding the impact of parasites on their health, production, and anthelmintic resistance.	We agree with this comment. We have added information about the impact of intestinal parasitism on horses' health and anthelmintic resistance in this species. (references added: 4,6, 9,13)
Page 4, Lines 82-84 – I don't see the need of inserting here this citation regarding a protozoan, a blood parasite, from mice, when previously you mentioned the importance of food in host's resistance and tolerance to helminths. Therefore, I recommend to remove it, because it can also be confusing for the reader.	We agree with this suggestion, and we removed it.
Materials and Methods<br>*Page 9*<br>Lines 181-182 – It is not clear the way you collect the L3 from the cultures: the Petri dishes were washed? The Whatman filter paper with culture are inverted and placed in a Baermann apparatus? Please be more specific.	We explained this method with more detail in the manuscript (line 181-182 in the revised manuscript with track changes).

Results<br>Pages 20, Line 428 – Instead of "…Volatil Fatty Acids…", write "…Volatile Fatty Acids…".	Corrected.
Discussion<br>Page 25, Line 551 – Instead of "…infesting larvae…", you should write "…infective larvae…".	Corrected.
Limitations<br>Page 26 – You should give a perspective of an ideal or optimal timespan for a longer field trial like this using a Latin Square Design to assess the same factors in horse parasitism level, both for each period and for the whole extent of the trial, eg., 2 months instead of 21 days and 1 year, instead of 4 months.	Thank you for this suggestion. We have included a perspective in that direction from lines 587 to 589 in the revised manuscript with track changes.
It would be interesting also to mention the economical part of such an important set of experiments like these ones you performed with horses, because I feel that available animals and funding is crucial for such a complex study like the one you did. Therefore, perhaps it would be important that you could share your thoughts on this matter, namely if the industry connected with horse production and private horse stud farms should be more engaged in funding this sort of research that is applied, but at the same time fundamental, regarding horse's health and welfare.	Following the referee's suggestion, we have included our thoughts on the importance of the funding of this type of experiments in our "Conclusions and Future perspectives" from line 609 to 612 in the revised manuscript with track changes.
Conclusions<br>Page 26, Line 590 – Instead of "…level of infection of the pasture…", write "…level of the pasture contamination…". The grass/herbage, does not get infected, but just contaminated.	Corrected.
Page 27, Lines 596 – 602 – Although your statements here are very important, this is not a direct conclusion from your work, therefore I advise one of two things: a) Change the title to Conclusions and Future perspectives; or b) Create a new title after the Conclusions one for Future perspectives and besides other thoughts, move the ones between lines 596 and 602 to this new chapter.	The title has been changed to 'Conclusions and Future Perspectives'.
References<br>Check if all Latin names, whether from parasites or hosts, are well written and/or in italic, because they must. See for instance references 7 and 8.	Done.

Reviewer #2

The Manuscript entitled: “Diet modulates strongyle infection and microbiota in the large intestine of horses” is interesting, fits the journal's profile but needs some explanations.

**Referees’ comments**	**Authors’ answers**
In Introduction is a lack of hypothesis of the study, please add.	We agree with this comment, and we have included our predictions at the end of the introduction from line 120 to line 123 in revised manuscript with track changes.
I suggest moving the information from l.111 – l.115 to another place in the text, maybe to Material and Methods.	We have merged this paragraph with the predictions at the end of the introduction, which helps understanding the aims of our study.
Was the nutritional value of the feed analyzed or are these values calculated (Table 1)?	The nutritional value of each raw material was analyzed by Dairy One laboratory (Ithaca, USA). We added this information to line 158 in the revised manuscript with track changes.
Please provide detailed information on which microscope the measurements were taken.	We have added the microscope references in the revised manuscript with track changes at lines 177, 184, and 258.
What was the internal standard used for the VFAs analyses? What column was used, please provide precise parameters? What was the carrier gas used?	We have added this information from line 237 to 240 in the revised manuscript with track changes.
In the discussion, I would suggest more references to current literature.	The following references have been added to provide more references to current literature: 48, 54, 56, 57, 58, 59, 60, 61, 62, 65 and 66.
